# Supplementary material for: AAV-Mediated Targeting of the Activin A-ACVR1R206H Signaling in Fibrodysplasia Ossificans Progressiva
Source: Biomolecules. 2023 Sep 8;13(9):1364. doi: 10.3390/biom13091364 (PMC10526456; doi:10.3390/biom13091364)
Supplement: Supplementary file 1 [file biomolecules-13-01364-s001.zip › biomolecules-13-01364-s001.pdf]

AAV-Mediated Targeting of the Activin A-ACVR1^{R206H} Signaling in Fibrodysplasia Ossificans Progressiva

Yeon-Suk Yang [1,†], Chujiao Lin [1,†], Hong Ma [2,3,4], Jun Xie [2,3,4], Frederick S. Kaplan [5,6,7], Guangping Gao [2,3,4,8,*] and Jae-Hyuck Shim [1,2,8,*]

[1] Department of Medicine, Division of Rheumatology, University of Massachusetts Chan Medical School, Worcester, MA 01655, USA; yen.yang@umassmed.edu (Y.-S.Y.), chujiao.lin@umassmed.edu (C.L.)
[2] Horae Gene Therapy Center, University of Massachusetts Chan Medical School, Worcester, MA 01655, USA; hong.ma2@umassmed.edu (H.M.), jun.xie@umassmed.edu (J.X.)
[3] Department of Microbiology and Physiological Systems, University of Massachusetts Chan Medical School, Worcester, MA 01655, USA
[4] Viral Vector Core, University of Massachusetts Chan Medical School, Worcester, MA 01655, USA
[5] Department of Orthopaedic Surgery, The Perelman School of Medicine at the University of Pennsylvania, Philadelphia, PA 19104, USA; frederick.kaplan@pennmedicine.upenn.edu
[6] Department of Medicine, The Perelman School of Medicine at the University of Pennsylvania, Philadelphia, PA 19104, USA
[7] The Center for Research in FOP and Related Disorders, The Perelman School of Medicine at the University of Pennsylvania, Philadelphia, PA 19104, USA
[8] Li Weibo Institute for Rare Diseases Research, University of Massachusetts Chan Medical School, Worcester, MA 01655, USA
* Correspondence: guangping.gao@umassmed.edu (G.G.); jaehyuck.shim@umassmed.edu (J.-H.S.).
† These authors contributed equally to this work.

Supplementary information

- Supplementary Figures S1-S4
- Supplementary Table S1

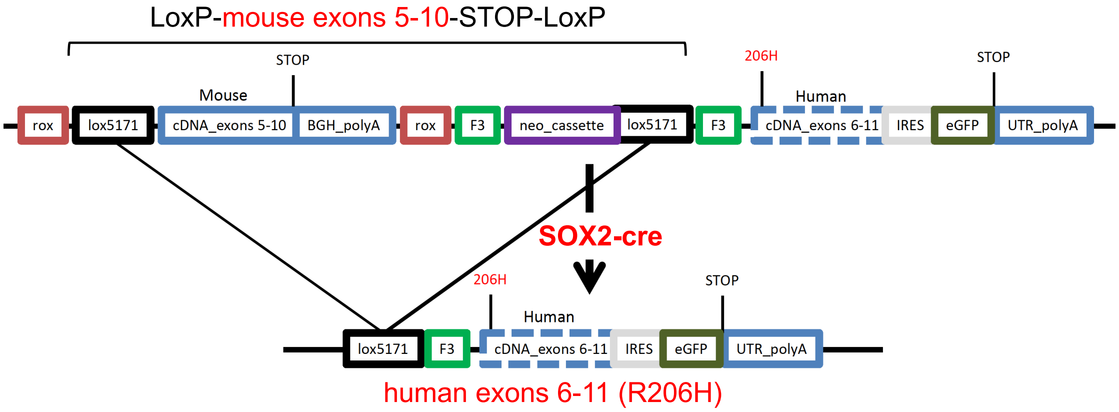

Supplementary Figure S1. Diagram showing the targeting construct to generate mice harboring a conditional knock-in allele of human *Acvr1*^{R206H} (*Acvr1*$^{R206H/+}$)

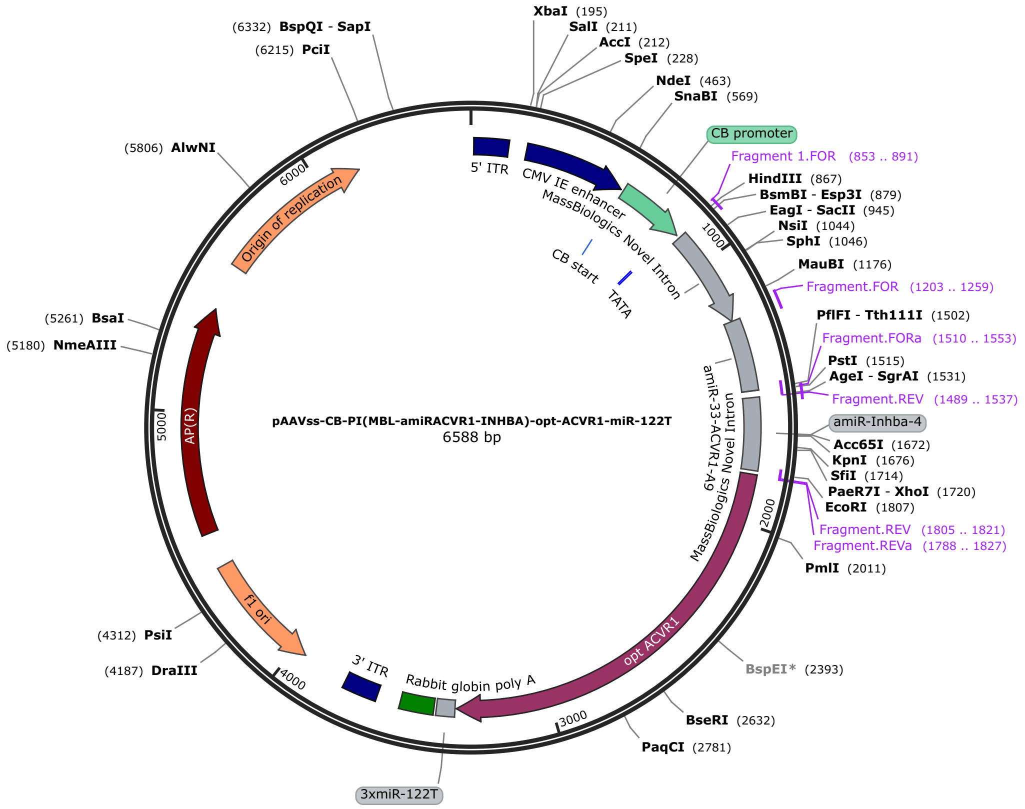

Supplementary Figure S2. Construction of AAV vector genome.

AAV vector genomes containing CBA (CMV enhancer/chicken β-actin) promoter, MBL intron (MassBiolocis), amiR-ACVR1^{R206H}, amiR-Inhba #4, ACVR1^{opt}, three tandem miR-122-TS (target sequence), and poly A sequences.

Supplementary Table S1: Sequences of primers and gblocks

1. Codon-optimized ACVR1 (ACVR1^{opt})

atggtcgatggagtgatgatcctgcctgtcctgattatgattgccctgcccagccccagcatggaagatgaaaaacctaaagtcaaccctaagctgtatatg
tgcgtgtgcgagggcctgagctgcggaaacgaggatcactgcgagggccagcagtgtttcagctccctgtccatcaatgacggcttccacgtgtaccaga
agggctgctttcaggtgtatgagcagggcaagatgacctgtaagacaccaccttccccaggacaggcagtggagtgctgtcagggcgattggtgtaacc
ggaatatcaccgcccagctgccaacaaagggcaagtctttccccggcacacagaactttcacctggaagtgggcctgatcatcctgagcgtggtgttcgcc
gtgtgcctgctggcatgtctgctgggagtggccctgagaaagtttaagcggagaaaccaggagcggctgaatccaagagatgtggagtacggcaccat
cgagggcctgatcaccacaaatgtgggcgactctacactggccgacctgctggatcacagctgcaccagcggctccggatctggcctgccctttctggtgc
agaggaccgtggcccggcagatcaccctgctggagtgcgtgggcaagggccggtacggagaagtgtggagaggatcctggcagggagagaacgtg
gcagtgaagatcttctctagccgggatgagaagtcttggtttagagagacagagctgtataacacagtgatgctgaggcacgagaatatcctgggcttca
tcgcctccgacatgacctctcgccactcctctacacagctgtggctgatcacccactaccacgagatgggctccctgtacgattacctccagctgaccacact
ggacacagtgtcttgcctgcggatcgtgctgtctatcgccagcggcctggcacacctgcacatcgagatctttggaacccagggcaagccagcaatcgca
cacagagatctgaagtctaagaacatcctggtgaagaagaatggccagtgctgtatcgccgatctgggcctggccgtgatgcacagccagtccaccaac
cagctggacgtgggcaacaatcctcgggtgggcacaaagagatacatggccccagaggtgctggatgagacaatccaggtggactgcttcgatagcta
taagagggtggacatctgggcctttggcctggtgctgtgggaggtggcaaggaggatggtgagcaacggcatcgtggaggactacaagccacccttct
atgacgtggtgcctaatgatccatcctttgaggacatgcgcaaggtggtgtgcgtggatcagcagaggcccaacatccctaatcgctggttcagcgacccc
accctgacatccctggccaagctgatgaaggagtgttggtatcagaatcctagcgccaggctgaccgccctgcgcatcaagaaaactctgactaaaatcg
acaatagcctggataaactgaaaaccgactgctga

2. amiR-ACVR1^{R206H}

agggctctgcgtttgctccaggtagtccgctgctcccttgggcctgggcccactgacagccctggtgcctctggccggctgcacacctcctggcgggcagc
tgtgtgtaatctggtgagccactgttgttctggcaatacctgacagtggcagatcagattacacacggaggcctgccctgactgcccacggtgccgtggcc
aaagaggatctaagggcaccgctgagggcctacctaaccatcgtggggaataaggacagtgtcaccc

3. amiR-INHBA #1

ggcagccttggagtgggttcctgccccctcgggcacacaaacagagctgaagaccaccctgggcacctccttggctggccgcatacctcctggcgggca
gctgtgtctttcttcttcttcttgccctgttctggtggtacccagggcaagatcaggaagaaagacacagaggcctgcctggccctcgagagactgccctga
ctgaaggccctatcaggtgggggaggggatcctgatagagggcactgctgccactgttggggcccaag

4. amiR-INHBA #2

ggcagccttggagtgggttcctgccccctcgggcacacaaacagagctgaagaccaccctgggcacctccttggctggccgcatacctcctggcgggca
gctgtgtttgccactgtcttctctggatgttctggtggtacccatccagagatcatagtggcaaacacagaggcctgcctggccctcgagagactgccctga
ctgaaggccctatcaggtgggggaggggatcctgatagagggcactgctgccactgttggggcccaag

5. amiR-INHBA #3

ggcagccttggagtgggttcctgccccctcgggcacacaaacagagctgaagaccaccctgggcacctccttggctggccgcatacctcctggcgggca
gctgtgttcctttcaagtatctctccttgttctggtggtacccaaggagagaatcctgaaaggaacacagaggcctgcctggccctcgagagactgccctga
ctgaaggccctatcaggtgggggaggggatcctgatagagggcactgctgccactgttggggcccaag

6. amiR-INHBA #4

ggcagccttggagtgggttcctgccccctcgggcacacaaacagagctgaagaccaccctgggcacctccttggctggccgcatacctcctggcgggca
gctgtgtgatctccgaggtctgctccatgttctggtggtacccatggagcagtgcccggagatcacacagaggcctgcctggccctcgagagactgccctg
actgaaggccctatcaggtgggggaggggatcctgatagagggcactgctgccactgttggggcccaag

7. amiR-INHBA #5

ggcagccttggagtgggttcctgccccctcgggcacacaaacagagctgaagaccaccctgggcacctccttggctggccgcatacctcctggcgggca
gctgtgttttgatgatgttttgaccattgttctggtggtacccaatggtcaattcgtcatcaaaacacagaggcctgcctggccctcgagagactgccctgact
gaaggccctatcaggtgggggaggggatcctgatagagggcactgctgccactgttggggcccaag

8. amiR-INHBA #6

Ggcagccttggagtgggttcctgccccctcgggcacacaaacagagctgaagaccaccctgggcacctccttggctggccgcatacctcctggcgggca gctgtgtctttcttcttcttcttgccctgttctggtggtacccagggcaagatcaggaagaaagacacagaggcctgcctggccctcgagagactgccctga ctgaaggccctatcaggtgggggaggggatcctgatagagggcactgctgccactgttggggcccaag

9. miR-122 target sequences

acaaacaccattgtcacactccaacaaacaccattgtcacactccaacaaacaccattgtcacactcca

10. Sequences of qPCR primers

Gene	Forward	Reverse
ACVR1^{opt}	GACTACAAGCCACCCTTCTATG	ACCAGCGATTAGGGATGTTG
ACVR1^{R206H}	TGGTACAAAGAACAGTGGCTTA	CCATACCTGCCTTTCCCGA
Egfp	AGCAAAGACCCCAACGAGAA	GGCGGCGGTCACGAA
Mouse *Inhba*	GGAGATAGAGGACGACATTGGC	ACGCTCCACTACTGACAGGTCA
Mouse *Sox9*	CACACGTCAAGCGACCCATGAA	TCTTCTCGCTCTCGTTCAGCAG
Mouse *Id1*	ACATGAACGGCTGCTACTCAC	GACTTCAGACTCCGAGTTCAGC
Mouse *Msx2*	GGAACTGGAAAAGCTGAAAATG	CACAGGTCTATGGAAGGGGTAG
Mouse *Tnalp*	CACAATATCAAGGATATCGACGTGA	ACATCAGTTCTGTTCTTCGGGTACA
Mouse *Osx*	ATGGCGTCCTCTCTGCTTGA	GAAGGGTGGGTAGTCATTTG
Mouse *Bglap*	GCAGCACAGGTCCTAAATAG	GGGCAATAAGGTAGTGAACAG
Mouse *Ibsp*	CAGGGAGGCAGTGACTCTTC	AGTGTGGAAAGTGTGGCGTT
Mouse *Col1α1*	ACTGTCCCAACCCCCAAAG	ACGTATTCTTCCGGGCAGAA
Mouse *Acbt*	AGGGAAATCGTGCGTGACAT	GGGCAATAAGGTAGTGAACAG
Mouse *Gapdh*	ACAGTCCATGCCATCACTGCC	GCCTGCTTCACCACCTTCTTG
Human *INHBA*	GGATGACATTGGAAGGAGGGCA	ACTGACAGGTCACTGCCTTCCT

Figure 2A

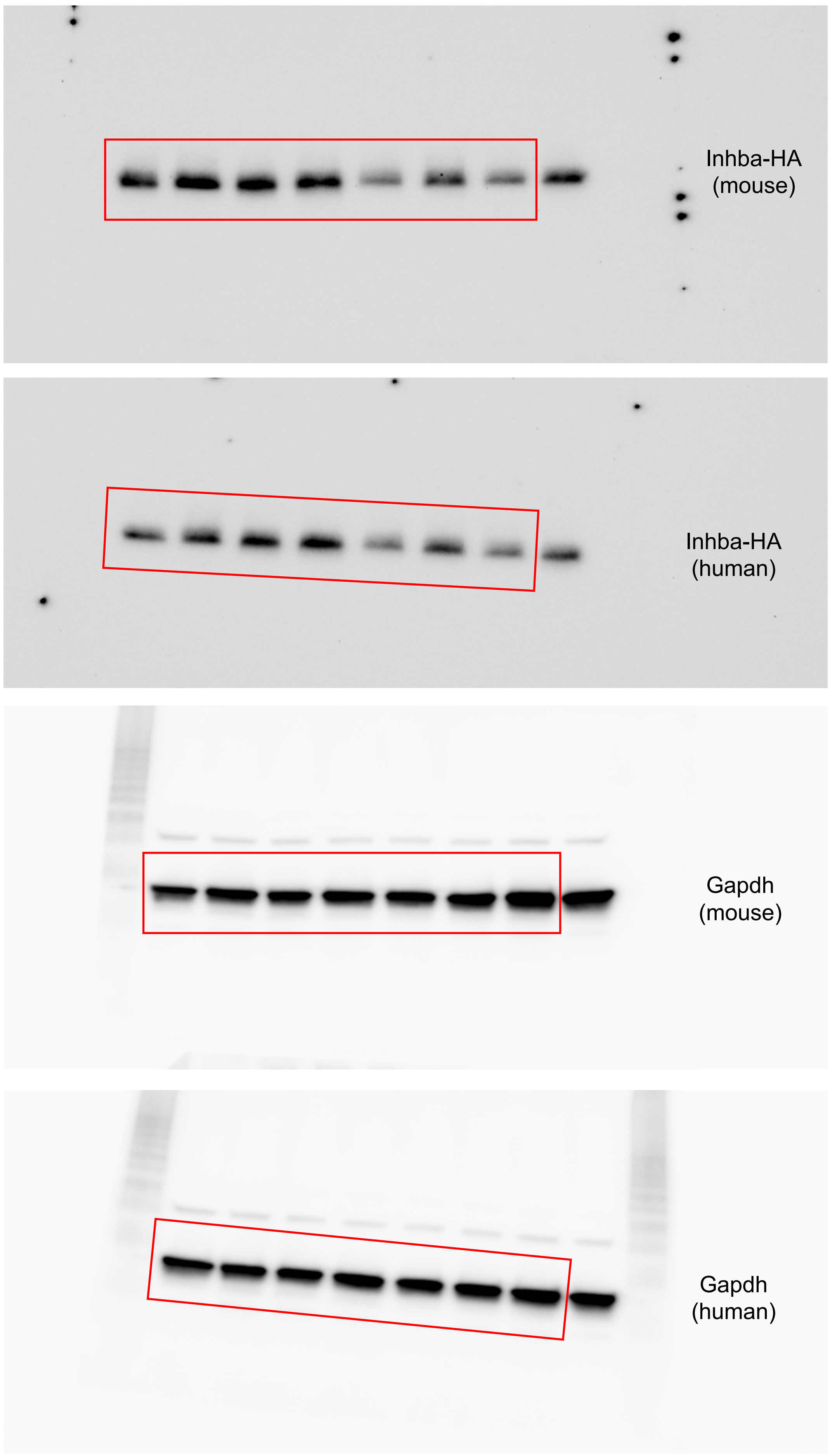

Figure S3: Original images of Figure 2A.

Figure 4G

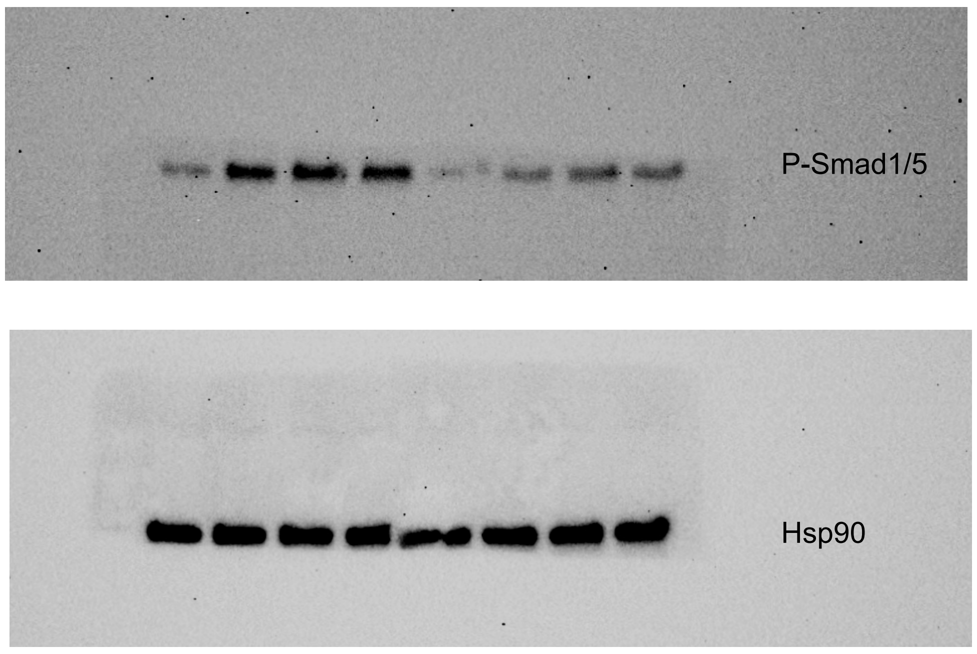

Figure S4: Original images of Figure 4G.
